# Overcoming PARP inhibitor resistance in ovarian cancer: what are the most promising strategies?

**DOI:** 10.1007/s00404-020-05677-1

**Published:** 2020-08-24

**Authors:** Daniel Martin Klotz, Pauline Wimberger

**Affiliations:** 1 Dresden and German Cancer Research Center (DKFZ), German Cancer Consortium (DKTK), Heidelberg, Germany; 2 Department of Gynecology and Obstetrics, Medical Faculty, University Hospital Carl Gustav Carus, Technische Universität Dresden, Dresden, Germany; 3 National Center for Tumor Diseases (NCT), Partner Site Dresden, Dresden, Germany

**Keywords:** Ovarian cancer, PARP inhibitors, Drug resistance, Clinical trials, Drug targets

## Abstract

**Purpose:**

Ovarian cancer is the most lethal gynaecological malignancy. Despite the introduction of bevacizumab, standard chemotherapy has remained largely unchanged and the vast majority of patients will relapse within the first two years of diagnosis. However, results from recent clinical trials demonstrating clinical benefits of PARP inhibitor treatment are rapidly changing therapeutic options for many patients with ovarian cancer.

**Methods:**

Given the introduction of new therapeutic options in the treatment of ovarian cancer, we critically review key clinical trials, areas of scientific research and its clinical relevance.

**Results:**

Most notably, patients with BRCA1/2 mutant ovarian cancer benefit from maintenance treatment with PARP inhibitors after (complete or partial) response to platinum-based chemotherapy. Here, we discuss the mechanism of PARP inhibition, multiple drug resistance mechanisms, including BRCA reverse mutations, altered PARP expression, changes in DNA repair pathways, kinase activation and additional drug targets that may augment PARP inhibition.

**Conclusion:**

Although the use of PARP inhibitors is a huge step forward, it is apparent that patients, both with and without BRCA-mutant ovarian cancer, will eventually become resistant to PARP inhibitors. Therefore, novel combination therapies may enhance PARP inhibitor efficacy and overcome resistance mechanisms.

## Introduction

While being the fifth most common gynaecological malignancy, ovarian cancer is the leading cause of death from gynaecological malignancies; about 7500 women are newly diagnosed and about 5500 die from the disease in Germany each year [1]. Epithelial ovarian cancer accounts for about 90% of the disease [2], of which high-grade serous ovarian cancer (HGSOC) shows the lowest average 5-year survival rates of only about 40% for advanced stages of the disease [3, 4]. The mainstay of treatment consists of surgical debulking with macroscopic complete resection and platinum-based chemotherapy. It has been demonstrated that surgical debulking is the only modifiable prognostic factor after diagnosis of HGSOC. If macroscopic complete resection can be achieved, 5-year survival rates may improve from around 20% to up to 60% for advanced HGSOC [3]. However, most patients (80%) will relapse within the first 2 years [5]. Despite the burden of the disease, standard chemotherapy regimens have remained largely unchanged for decades [6, 7].

However, the most notable exception to the dearth of new treatment options has been the introduction of the anti-angiogenesis drug bevacizumab for patients with advanced ovarian cancer. Until recently, it had been the only new approved therapy for the last decade. Maintenance therapy with bevacizumab showed an improved PFI [8, 9]. Although it failed to reduce overall survival in the ITT population, the subgroup analysis of high-risk patients showed an overall survival benefit, with high-risk being FIGO stage III with > 1 cm residual disease, FIGO stage IV or inoperable disease [8]. The other very recent milestone and most intriguing new therapeutic option consists of polyADP-ribose polymerase inhibitors (PARPis). In the last few years, several clinical trials have shown benefits for primary as well as recurrent ovarian cancer when used as maintenance treatment after initial complete response (CR) or partial response (PR) to platinum-based chemotherapy [10–18] (Table 1). This can partially be explained by the high prevalence of tumours with homologous recombination deficiency (HRD), which is found in about 50% of all ovarian cancers. BRCA1/2 deficiency accounts for about 20% of these cases [19]. This underlies the molecular rationale and the significant clinical benefit of PARPis in BRCA1/2 mutation carriers and patients with HR-deficient tumours. Although it also shows a significant prolonged PFS in patients with BRCA1/2wt and HR-proficient tumours, this substantial clinical benefit is less compared to the other subgroups mentioned above [14]. Due to the lack of a widely available (EMA approved) HRD test with a reproducible threshold, one of the main clinical challenges is the identification of patients that are most likely to benefit from PARP inhibitors. This is particularly true for patients with BRCA1/2wt tumours. Although the most recent first-line clinical trials in ovarian cancer used the same HRD test (myChoice^®^, Myriad Genetics), thresholds to define patients’ tumours as HRD differed, which greatly hinders comparison of clinical response and prevents smooth translation into common clinical practice [10–12]. Nonetheless, this HRD test has been FDA-approved to determine HRD status in tumours.

 It is critical to understand the underlying mechanisms of resistance to enhance the clinical use of PARP inhibition in ovarian cancer. Here, we discuss the mechanisms of PARP inhibition, potential drug resistance mechanism, and strategies to enhance efficacy of PARPis in the setting of ovarian cancer. Given the new therapeutic options in the treatment of ovarian cancer, we critically review key areas of scientific research and its clinical relevance.

## The role of PARP

The enzyme PARP belongs to a family of proteins that catalyse the polymerisation of ADP-ribose (PARylation) to its target proteins or itself [20]. PARylation uses NAD + as a substrate, releasing nicotinamide as the by-product. PARP-1 and PARP-2 are the only members of the family that have been shown to be involved in DNA single-strand break (DNA SSB) repair [21]. Other functions include cell death and cell cycle regulation [20].

PARP-1 is the most studied protein of the family and contains multiple domains with well-characterised functions, including binding to DNA break sites, nuclear recruitment and the enzymatic domain [20]. Specifically, PARP-1 is upstream of the DNA SSB repair mechanism called base excision repair [21]. This allows the identification of a damaged part of DNA, followed by the synthesis of new DNA at the site of the DNA SSB. Here, PARP-1/2 act as a sensor for DNA SSBs, recruiting other key players, such as XRCC1 and DNA ligase 3 [20]. Typically, DNA SSBs are repaired in G1. If unsuccessful, DNA double strand breaks (DNA DSBs) accumulate upon DNA replication [22]. Cells have evolved mechanisms to repair such DNA DSBs, namely HR and non-homologous end-joining (NHEJ). HR and NHEJ are important to maintain genomic integrity, but differ significantly in their mechanism, accuracy, and time of repair within the cell cycle. HR acts mainly in S phase after DNA replication, because it requires DNA sequence homology of sister chromatids as a template to repair DNA DSBs [23]. It is initiated upon DNA 5′-end resection and demonstrates a high-fidelity mechanism that conserves genomic stability. Key players are BRCA1/2, RAD51, and PALB2 [24]. NHEJ acts throughout the cell cycle by directly ligating the ends of DNA DSBs, which increases the chance of deletions and mutations since sister chromotids are not used as template [24]. An important player for NHEJ is 53BP1 which acts in combination with other factors in determining DNA DSB repair [25].

## The mechanism(s) of PARP inhibitors

PARPis compromise adequate DNA SSB repair, leading to the persistence of DNA SSBs. If unrepaired in G1, this will lead to the accumulation of DNA DSBs upon DNA replication during S phase. The mechanism of action of PARPis is as follows: PARPis block the NAD + binding site on PARP, effectively inhibiting PARylation. This prevents PARP dissociation from DNA SSBs, resulting in both accumulation of unrepaired DNA SSBs and PARP trapping [26]. If the DNA SSB is not fully repaired and the cell enters S phase, DNA DSBs occur, which, in turn, require an effective repair mechanism to ensure genomic stability [26, 27]. Therefore, cells rely disproportionally on DNA DSB repair in the presence of PARP inhibition. In the case of HRD, as seen in BRCA1/2 mutations carriers, cells repair their DNA DSBs via NHEJ, resulting in deleterious genomic instability. This mechanism is termed synthetic lethality, because PARPis exploit the Achilles’ heel of BRCA1/2-deficient tumour cells. Additionally, PARP trapping occurs because of the lack of autoPARylation which normally initiates DNA dissociation of PARP [28]. Interestingly, the most commonly used PARPi show different potencies of PARP trapping, which is (partly) independent of the inhibitory effect of PARP [26–28]. However, it has been shown that the exact extent of PARP trapping potency depends on both the experimental method and whether it is tested as monotherapy or combination therapy, making comparisons difficult. Typically, talazoparib commonly shows the greatest potency and veliparib the lowest, with olaparib, niraparib, and rucaparib in-between [28–30]. In summary, PARPis exhibit two main mechanisms of action, by compromising DNA SSB repair and by PARP trapping. Many clinical trials have shown the efficacy of PARPi in patients with ovarian cancer [10–18].

## Key clinical trials of PARP inhibitors in ovarian cancer

PARPis are licensed as maintenance treatment for recurrent ovarian cancer after response (CR/PR) to platinum-based chemotherapy independent of BRCA1/2 status and also as maintenance treatment for primary high-grade serous ovarian, fallopian tube, or peritoneal cancer after response (CR/PR) to platinum-based chemotherapy in BRCA1/2 mutation carriers. In addition, rucaparib is also licenced as a monotherapy in platinum-sensitive relapsed/progressive BRCAm (somatic or germline) ovarian cancer, if the patient had ≥ 2 lines of platinum-based chemotherapy and is unable to tolerate another platinum-based chemotherapy. There have been many recent clinical trials assessing the efficacy of PARPis in patients with recurrent or primary ovarian cancer [10–18]. This showed that PARPis significantly prolong the PFS as a maintenance treatment after response (CR/PR) to platinum-based chemotherapy, most notably in patients with BRCAm and/or HR-deficient ovarian cancers (Table 1).

**Table 1:** Key clinical trials showing clinical efficacy of PARP inhibitors in ovarian cancer

Clinical trial/phase	Setting	Treatments	Patients (randomised)	Results median PFS in months (with 95% CI)				Hazard ratio (95%CI), *p* value	References
SOLO-1 III	Newly diagnosed histologically confirmed advanced high-grade serous or endometrioid ovarian cancer, primary peritoneal cancer, or fallopian-tube cancer, FIGO stage III or IV, deleterious or suspected deleterious germline or somatic *BRCA1/2* mutation, after 6–9 cycles of platinum-based chemotherapy with CR or PR, Surgery (primary or interval) allowed, no bevacizumab	Olaparib versus Placebo	*n* = 391	Olaparib arm All (*n*=260) NR (at time of publication)		Placebo arm All (*n* = 131) 13.8		HR 0.30 (0.26–0.41), *p *< 0.001	[13]
PAOLA-1 III	Newly diagnosed high-grade serous or endometrioid ovarian cancer fallopian tube or primary peritoneal cancer, FIGO stage III or IV, minimum of six and max. of nine cycles of platinum-taxane based chemotherapy with NED/CR/PR, maintenance with bevacizumab and a minimum of three cycles of bevacizumab with last three cycles of chemotherapy Surgery (primary or interval) allowed, HRD testing with myChoice^®^ HRD Plus, Myriad Genetics	Olaparib plus Bevacizumab versus Placebo plus Bevacizumab	*n* = 806	Olaparib arm All (*n* = 537) 22.1 BRCAm (*n* = 157): 37.2 HRDpos, incl. BRCAm (*n* = 255) 37.2 HRD-pos, BRCAwt (*n* = 97): 28.1 BRCAwt+HRD-neg/unknown) (*n* = 282) 16.9		*Placebo arm * All (*n* = 269) 16.6 BRCAm (*n* = 80): 21.7 HRDpos. incl. BRCAm (*n* = 132) 17.7 HRD-pos. BRCAwt (*n* = 55): 16.6 BRCAwt+HRD-neg/unknown) (*n* = 137) 16.0		HR 0.59 (0.49–0.72), *p *< 0.0001 HR 0.31 (0.20–0.47) HR 0.33 (0.25–0.45) HR 0.43 (0.28–0.66) HR 0.92 (0.72–1.17)	[11]
PRIMA III	Newly diagnosed primary histologically confirmed advanced cancer of the ovary, peritoneum, or fallopian tube, FIGO stage III required visible residual tumour after primary surgery (unless interval surgery) or FIGO IV, a minimum of four cycles of platinum-based chemotherapy with CR/PR, Surgery (primary or interval) allowed, HRD testing with myChoice^®^, Myriad Genetics	Niraparib versus Placebo	All (*n* = 799) HRDpos (*n* = 373)	Niraparib arm All (*n* = 487): 13.8 (11.5–14.9) HRDpos (*n* = 247): 21.9 (19.3-NE) HRDpos+ BRCAm (*n* = 152) 22.1 (19.3–NE) HRDpos +BRCAwt (*n* = 95) 19.6 (13.6–NE) HR-proficient (*n* = 169): 8.1 (5.7–9.4)		Placebo arm All (*n* = 246): 8.2 (7.3–8.5) HRDpos (*n* = 126): 10.4 (8.1–12.1) HRDpos+ BRCAm(*n* = 152) 10.9 (8.0–19.4) HRDpos +BRCAwt (*n* = 55) 8.2 (6.7–16.8) HR-proficient (*n* = 80): 5.4 (4.0–7.3)		HR 0.62 (0.5–0.75), *p *< 0.0001 HR 0.43 (0.31–0.59), *p *< 0.0001 HR 0.40 (0.27–0.62), *p *< 0.001 HR 0.50(0.31–0.83), *p *= 0.006 HR 0.68(0.49–0.94), *p *= 0.020	[12]
VELIA III	Newly diagnosed primary, histologically confirmed, high-grade serous epithelial ovarian, fallopian tube, or primary peritoneal carcinoma, FIGO stage III or IV, a minimum of six cycles of carboplatin/Paclitaxel chemotherapy, Surgery (primary or interval) allowed, HRD testing with myChoice® CDx or BRACAnalysis CDx, Myriad Genetics	Veliparib throughout (during chemotherapy and maintenance) (V-throughout) versus Veliparib during chemotherapy with placebo maintenance (VCTXonly) versus Placebo throughout chemotherapy and as maintenance)	All (*n* = 1140) V-throughout (*n* = 375) VCTX-only (*n* = 383) Placebo (*n* = 382)	Veliparib throughout arm ITT (*n* = 382) 23.5 (19.3–26.3) HRDpos (*n* = 214) 31.9 (25.8–38.0) BRCAm *n* = 108) 34.7 (31.8–xx)	Veliparib during CTx only arm ITT (*n* = 383) 15.2 (14.1–17.3) HRD (*n* = 206) 18.1 (16.4–22.7) BRCAm *n* = 98) 21.1 (17.0–25.5)		Placebo arm ITT (17.3 ( 15.1–19.1) HRDpos (*n* = 207) 20.5 (17.8–22.8) BRCAm (*n* = 92) 22,0 (17.8–29.1) ITT (*n* = 375) 17.3 (15.1–19.1) HRD (*n* = 207) 20.5 (17.8–22.8) BRCAm (*n* = 92) 22.0 (17.8–29.1)	HR 0.68 (0.56–0.83), *p *< 0.001 HR 0.57 (0.43–0.76), *p *< 0.001 HR 0.44 (0.28–0.68), *p *< 0.001 HR 1.07 (0.90–1.29) HR 1.10 (0.86–1.41) HR 1.22 (0.82–1.80)	[10]
Study 19 II	Recurrent platinum-sensitive high-grade serous ovarian or fallopian-tube cancer or primary peritoneal cancer, at least two courses of platinum-based chemotherapy, (a minimum of four cycles) in last platinum-based chemotherapy with CR/PR	Olaparib (capsules) versus Placebo	*n* = 265	Olaparib arm All (*n* = 136) 8.4 (7.4–11.5) BRCAwt (*n* = 57) 7.4(5.5–10.3) BRCAm (*n* = 74) 11.2 (8.3–NC)		Placebo arm All(*n* = 129) 4.8 (4.0–5.5) BRCAwt (*n* = 61) 5.5 (3.7 -5.6) BRCAm(*n* = 62) 4.3 (3.0–5.4)		HR 0.35 (0.25–0.49), *p *< 0.0001 HR 0.54 (0.34–0.85), *p *< 0.0075 HR 0.18 (0.10–0.31), *p *< 0.0001	[15, 31]
NOVA II	Recurrent platinum-sensitive ovarian cancer, fallopian tube cancer, or primary peritoneal cancer with predominantly high-grade serous histologic features, at least two courses of platinum-based chemotherapy, (a minimum of four cycles) in last platinum-based chemotherapy with CR/PR, HRD testing with myChoice^®^, Myriad Genetics	Niraparib versus Placebo	All (*n* = 553) Niraparib (*n* = 372) Placebo (*n* = 181)	Niraparib arm gBRCAm cohort (*n* = 138) 21.0 Non-gBRCA with HRD (*n* = 106) 12.9 Non-gBRCA cohort (*n* = 234) 9.3		Placebo arm gBRCAm cohort (*n* = 65) 5.5 Non-gBRCA with HRD (*n* = 56) 3.8 Non-gBRCA coh-ort (*n* = 116) 3.9		HR 0.27 ( 0.17–0.41), *p *< 0.001 HR 0.38 ( 0.24–0.59), *p *< 0.001 HR 0.45 (0.34–0.61)	[14]
SOLO-2 III	Recurrent, HGSOC or high-grade endometrioid cancer, including primary peritoneal or fallopian tube cancer in patients with a predicted or suspected deleterious somatic or germline *BRCA1/2* mutation, at least two courses of platinum-based chemotherapy, a mini of four cycles) in last platinum-based chemotherapy with CR/PR	Olaparib versus placebo	*n* = 295	Olaparib All (*n* = 196) 19.1 (16.3–25.7)		Placebo All (*n* = 99) 5.5 (5.2–5.8)		HR 0.30 (0.22–0.41), *p *< 0.0001	[16]
Ariel-2 (part 1) II	Recurrent high-grade serous or endometrioid ovarian, fallopian tube, or primary peritoneal carcinoma, at least one prior platinum-based chemotherapy, more than 6 months PFS after last platinum-based chemotherapy HRD (LOH) measured by NGS	Rucaparib	*n* = 194	BRCAm (*n* = 40) 12.8 (9.0–14.7) BRCAwt and high LOH (*n* = 82) 5.7 (5.3–7.6) BRCAwt and low LOH (*n* = 70) 5.2 (3.6–5.5)				HR 0.27 (0.16–0.44), *p *< 0.0001 (compared to BRCAwt and low LOH) BRCAwt and high LOH HR 0.62 (0.42 0.90), *p *= 0.011 (compared to BRCAwt and low LOH)	[17]
Ariel-3 II	Recurrent platinum-sensitive, high-grade serous or endometrioid ovarian, primary peritoneal, or fallopian tube cancer, at least two prior platinum-based chemotherapy, more than 6 months PFS after last platinum-based chemotherapy (CR/PR) before trial therapy, HRD (LOH) measured by NGS	Rucaparib versus Placebo	*n* = 564	Rucaparib arm ITT (*n* = 375) 10.8 (8.3–11.4) BRCAm (*n* = 130) 16.6 (13.4–22.9) HRD (*n* = 236) 13.6 (10.9–16.2)		Placebo arm ITT(*n* = 189) 5.4 (5.3–5.5) BRCAm (*n* = 66) 5.4 (3.4–6.7) HRD (*n* = 118) 5.4 (5.1–5.6)		HR 0.36 (0.30–0.45) HR 0.23 (0.16–0.34), *p *< 0.0001 HR 0.32 (0.24–0.42), *p *< 0.0001	[18]

Olaparib was one of the first PARPis, which was introduced into clinical practice for (recurrent) ovarian cancer. The randomised, double-blind phase 2 clinical trial with olaparib (Study 19) showed an improved PFS of 8.4 months versus 4.8 months in patients with platinum-sensitive recurrent ovarian cancer (Table 1) [15]. The further subgroup analysis of BRCA mutation carriers (about 50% of the randomised patients) showed a significantly improved PFS of 11.2 versus 4.3 months (HR 0.18 [95% CI 0.10–0.31], *p* < 0.0001) [15, 31]. The overall survival benefit was most pronounced in patients with BRCA1/2 mutations with a median of 34.9 months (29.2–54.6 months) versus 30.2 months (23.1–40.7 months), HR 0.62 [95% CI 0.41–0.94], nominal *p* = 0.025) [32]. This did not meet the statistical significance threshold set in this clinical trial, but demonstrated that BRCA1/2 mutations carriers are the patients that most likely benefit from olaparib maintenance treatment after response (CR/PR) to platinum-based chemotherapy [32]. The randomised, double-blinded, placebo-controlled SOLO-2 trial confirmed these results in olaparib-treated patients with BRCA-mutated platinum-sensitive recurrent ovarian cancer, showing an improved PFS of 19.1 months versus 5.5 months (HR 0.30 [95% CI 0.22–0.41]) [16].

Another study (ENGOT-OV16/NOVA) evaluated the effect of niraparib maintenance treatment after response (CR/PR) to platinum-based chemotherapy. This randomised, double-blinded, placebo-controlled phase 3 clinical trial distinguished three groups of patients in the order of increasing efficacy to niraparib: BRCAwt and HR-proficient tumours < BRCAwt and HR-deficient tumours < BRCA1/2 m tumours [14]. This showed that patients with BRCA1/2 mutant tumours benefited most from maintenance treatment with 21.0 versus 5.5 months (HR 0.27 [95% CI 0.17–0.41]) (Table 1) [14]. Expanding the use of PARPis beyond BRCA mutations and trying to identify predictive biomarkers, the ARIEL-2 (part1) trial further classified tumours according to HRD status. The trial used high versus low loss of heterozygosity (LOH) as a marker, measured by NGS. Although showing an improved PFS in patients with high LOH BRCAwt tumours versus low LOH BRCAwt tumours [5.7 months (5.3–7.6 months) versus 5.2 months] (3.6–5.5 months) (HR 0.62 [95% CI 0.42–0.90]), this single-arm trial could not determine whether or not LOH/HRD status can be used as a predictive biomarker [17]. The use of HRD status as a biomarker for treatment response was further assessed in the double-blind, placebo-controlled, ARIEL-3 trial (phase 3) (Table 1) [18]. This trial also included an exploratory analysis showing that patients with BRCAwt and low LOH tumours have a significantly improved PFS when treated with rucaparib versus placebo (HR 0.58 [95% CI 0.40–0.85]) [18]. Together with the results of the NOVA and Study19 trial [14, 15], the ARIEL-3 trial expanded the use of PARPis to patients with platinum-sensitive recurrent ovarian cancer beyond HRD and BRCA mutation status [18].

These trials were followed by studies investigating the benefit in patients with newly diagnosed ovarian cancer after responding (CR/PR) to first-line platinum-based chemotherapy. A recent clinical trial (SOLO-1) compared olaparib versus placebo in BRCA1/2 mutation carriers with newly diagnosed ovarian cancer after response (CR/PR) to platinum-based chemotherapy [13]. This clinical trial (SOLO-1) showed a significantly improved PFS in the olaparib arm (60% versus 27%) at 3 years (Table 1) [13]. At the time of publication, the median PFS was not reached (i.e. not published) for the treatment arm and the clinical trial has not yet reported on overall survival. Interestingly, the authors estimate that the first subsequent therapy or death would be 51.8 months in the olaparib and 15.1 months in the placebo group (HR 0.30 [95% CI 0.22–0.40] [13]. The most recently published trials (VELIA, PRIMA, and PAOLA-1, Table 1) demonstrated a clear benefit of PARPis (veliparib, niraparib, and olaparib) in first-line treatment in patients with HR-deficient ovarian cancer, showing a significant improved PFS [10–12]. In addition to the different PARPis investigated as first-line therapy in these clinical trials, many key parameters also differed: combination treatment (bevacizumab or not), start time of the individual PARPi treatment (only during, throughout or after CR/PR to chemotherapy), patient cohort (high risk of relapse/progression or broad patient cohort), and thresholds for HRD testing of tumour tissue (myChoice^®^, Myriad Genetics). This requires careful consideration of when and how to translate these findings into common clinical practice (Table 1) [10–12]. First, the PAOLA-1 trial included bevacizumab in the maintenance treatment, which was part of the inclusion criteria and had to be started during chemotherapy; the other two trials did not. Given that bevacizumab maintenance is the standard treatment in many countries for advanced ovarian cancer, it would have been most interesting to investigate the clinical efficacy of combining of niraparib and bevacizumab in the patient cohort of the PRIMA trial. Interestingly, a three-arm clinical trial comparing niraparib and bevacizumab (± a checkpoint inhibitor) versus standard of care in patients with platinum-sensitive recurrent ovarian cancer is planned, but not yet recruiting (NCT03806049). This trial is based on the randomised superiority phase 2 trial of niraparib alone versus niraparib and bevacizumab in platinum-sensitive recurrent ovarian cancer, which showed an improved PFS of 11.9 months (8.5–16.7) versus 5.5 months (3.8–6.3), (HR 0.35 [95% CI 0.21–0.57], *p* < 0.0001) [33]. Second, the VELIA trial was the only trial that was designed with two different experimental arms: PARPi treatment during chemotherapy only, followed by placebo maintenance treatment compared to veliparib throughout chemotherapy and in the maintenance treatment. It demonstrated that PARP inhibition (with veliparib) during chemotherapy only is inferior to PARP inhibition throughout chemotherapy and maintenance treatment. The chemotherapy-only arm did not demonstrate an improved PFS across the different subgroups (BRCAm, HRD, and BRCAwt). The veliparib (during chemotherapy only)-treated ITT patient cohort showed a PFS of 15.2 months (14.1–17.3 months) versus 17.3 months (15.1–19.1 months) in the placebo-treated ITT patient cohort (HR 1.07 [95% CI 0.90–1.29]) [10]. Third, compared to the other two trials, the PRIMA trial included high-risk patients, i.e., FIGO III patients with visible residual tumour after primary debulking and inoperable FIGO IV disease [11]. This may explain the difference in the median PFS in the overall populations between the clinical trials: 23.8 months (VELIA) ≥ 22.1 months (PAOLA-1) > 13.8 months (PRIMA) [10–12]. The PRIMA trial was also the only trial (of the three mentioned above), which showed a significant benefit in PFS for niraparib-treated patients with HR-proficient tumours (incl. unknown HRD status), albeit to a smaller effect than seen in the HRD cohort. Specifically, niraparib significantly improved PFS in patients with HR-proficient tumours with 8.1 months (5.7–9.4 months) versus 5.4 months (4.0–7.3 months) in placebo-treated patients with HR-proficient tumours (HR 0.68 [95% CI 0.49–0.94], *p* = 0.020) [11]. Finally, the three clinical trials (VELIA, PRIMA, and PAOLA-1) relied on a commercially available assay to assess HRD status (myChoice^®^, Myriad Genetics); yet agreed standard procedures are lacking, i.e., the thresholds that define “HRD positivity” [10–12]. The set threshold for positive result of HRD was ≥ 42 in the PRIMA and PAOLA-1 trials, whereas the threshold was set as ≥ 33 in the VELIA trial, consequently, including more patients with HR-deficient tumours in the latter. Hence, one of the biggest challenges will be establishing clinically reproducible, widely available, and standardised testing for HRD status in the absence of BRCA1/2 mutations. This would identify those patients that will benefit from PARP inhibition after response to chemotherapy. A serological test would be of high clinical interest, if it circumvents the requirement of tissue sampling, and ultimately allowing the stratification of patients with recurrent ovarian cancer, when obtaining fresh tumour tissue is not typically an option.

Although these clinical trials showed promising results by significantly improving PFS, key questions remain. It is obvious that most, if not all, patients will eventually develop PARPi resistance. The time of resistance may dependent on the distinct vulnerability that underlies the efficacy of PARPis, i.e., BRCA mutation and HRD status. One could speculate that the degree of complexity of HRD in tumours underpins the development of resistance. This means that resistance to a PARPi would develop quicker if it does not “require” to counteract a (comparatively) complex mechanism. This assumption is based on the clinical observation that, after response to platinum-based chemotherapy, patients with tumours classed as “HR-proficient” benefit less from PARPis compared to patients with HR-deficient or BRCA1/2 mutant tumours [14]. Genomic analysis of long-term versus short-term responders to olaparib showed that response to olaparib was associated with BRCA1/2 mutations [34]. The authors further speculated that the underlying type of BRCA mutations may allow for a more accurate prediction of long-term responders. Similarly, a more accurate HRD test could give us a better understanding of the factors contributing to a sustained clinical response to PARP inhibition. On one hand, a potential approach to identify novel biomarkers would be to uncover the underlying resistance mechanisms in patients who do not respond to PARP inhibition. On the other hand, it would be essential to identify long-term survivors with BRCAwt and HR-proficient tumours treated with PARPi and characterise the underlying biomarkers that may predict such an exceptional response. This exciting avenue would also shed light onto potential strategies to enhance PARPi efficacy.

## Resistance mechanisms and strategies to increase efficacy

**Fig. 1:**
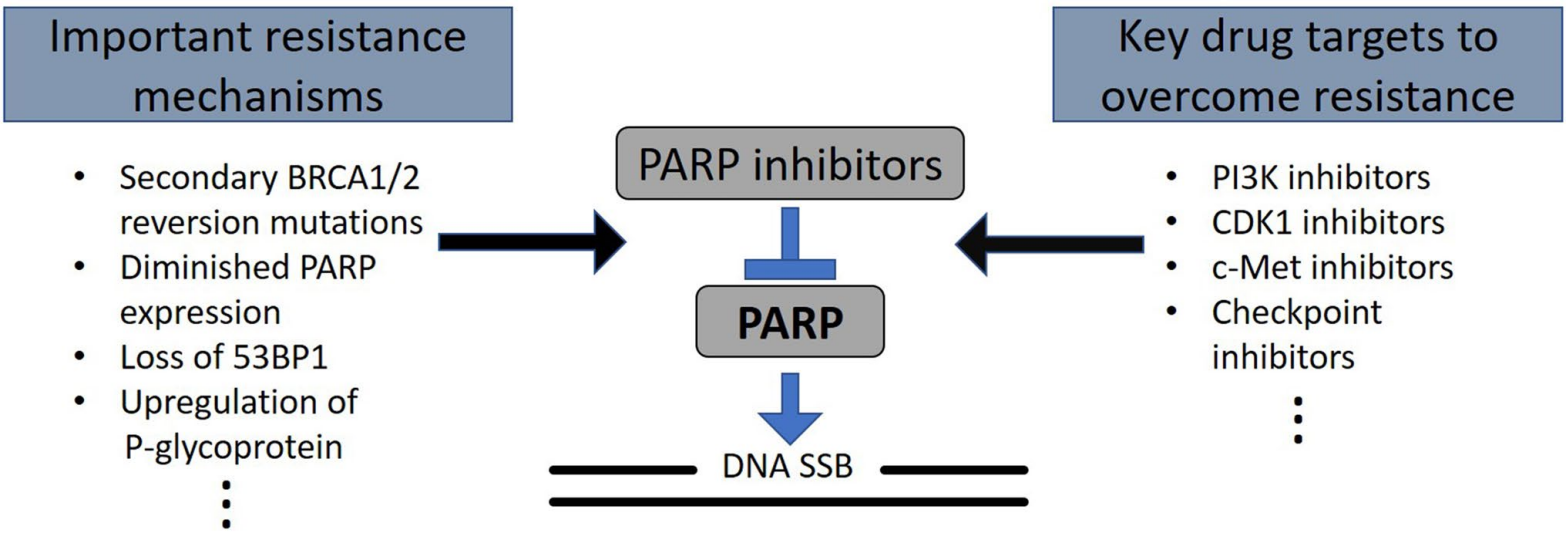
Resistance mechanisms and potential targets of combination therapies. A summary of resistance mechanisms and potential drug targets with a list of corresponding inhibitors that can be used in combination with PARPi

Many potential mechanisms of PARPi resistance have been described (Fig.1). The strongest rationale for the clinical development of PARPis stemmed from the response seen in BRCA1/2-deficient (and HR-deficient) cells. Hence, an intriguing mechanism of resistance involves secondary somatic reversion mutations in the BRCA1/2 genes in tumour cells, which essentially restore HR [35]. This could potentially be explained by tumour heterogeneity and clonal expansion driven by chemotherapy, termed “Darwinian escape” [36]. Comparing primary and recurrent ovarian cancers, it was shown that 13 out of 46 recurrent HGSOCs (28.3%, 95% CI 17.3–42.6%) had a secondary mutation and 2 out of 64 primary HGSOCs (3.1%, 95% CI 1.0–10.7%) in germline BRCA1/2 mutation carriers. This was even more pronounced in platinum-resistant versus platinum-sensitive ovarian cancer, with almost 50% of platinum-resistant cancer (12 out of 26) showing BRCA1/2 reversion mutations [37]. Secondary reverse mutations in BRCA1/2 were also shown to correlate with PARPi resistance in ovarian cancer and other cancers [35, 37]. Most interestingly, secondary reversion mutation of BRCA1/2 alleles have been detected by analysing circulating free DNA (cfDNA) in patients with prostate cancer [38]. This would offer a non-invasive method to more accurately predict response to platinum-based chemotherapy and/or PARPi treatment [38]. Other mechanisms affecting gene expression include BRCA1/2 hypermethylation [27]. Secondary reversion mutations of RAD51, a component of the HR machinery, have been implicated in causing  PARPi resistance in rucaparib-treated patients with platinum-sensitive HGSOC [39].

However, PARPi efficacy can also be impaired by BRCA-independent mechanisms that restore HR. The TP53-binding protein 1 (53BP1) is another important player in deciding the pathway by which DNA DSBs are repaired. It antagonises BRCA1 and inhibits end resection of DNA DSBs, thus favouring NHEJ [40]. In turn, the loss of 53BP1 has shown to favour HR in the absence of BRCA by potentially promoting DNA DSB end resection and RAD51 recruitment [41]. In tumour samples, low expression levels of 53BP1 correlated with a poorer response to PARPis in ovarian cancer with HRD [42]. In triple-negative breast cancer models, loss of 53BP1 rescues BRCA deficiency, reduces chemotherapy hypersensitivity, and is associated with a poorer prognosis [43]. This suggests clinical significance in testing for 53BP1 status in patients to assess efficacy of PARP inhibition. Similarly, the loss of a downstream factor of 53BP1, Rev7, has also shown to restore HR in BRCA-deficient cells, leading to PARPi resistance [44]. It remains to be seen how this mechanism of resistance could be alleviated. One could speculate that yet to be discovered factors that activate or stabilise 53BP1 could be used to restore drug efficacy in this context.

Another possibility is the loss or diminished expression of PARP-1 in tumour cells, because they essentially lack the drug target. This is particularly true for the mechanism of PARP trapping, which relies on the formation of DNA-bound complexes [26]. This suggests that stable PARP–DNA complex (at least) contributes to the cytotoxic effect of PARP inhibition [28]. Other resistance mechanisms are linked to the dynamic equilibrium between PARP-1 and PARG. The latter inhibits PARylation and effectively works as a physiological PARPi, which is needed to fully block PARylation in PARPi-treated cells [45].

Besides general drug efflux mechanisms involving p-glycoproteins [27], PARPi resistance could rely on other potentially ‘drugable’ factors that impair PARPi efficacy. Hence, PARPi sensitivity could be restored using combination therapy if the resistance mechanisms could be circumvented.

An interesting target could be the receptor tyrosine kinase c-Met, mesenchymal–epithelial transition factor, which is encoded by the c-met protooncogen. It is known that high c-Met expression is associated with a poor prognosis in ovarian cancer [46], which hints at a potential therapeutic exploitation of this pathway. More recently, c-Met has been linked to PARPi resistance in breast cancer cells, as well as restoring HR in a BRCA-independent manner to impair PARPi function. A small phase 2 clinical trial assessing the monoclonal antibody rilotumumab in ovarian cancer has shown no benefits as a single agent [47]. However, patient selection has been (at least) questionable, because c-Met expression has not been an inclusion criterion. Given that one patient had a complete response and two patients (out 31) had a 6-month PFS, it would be interesting to look for aberrant c-Met expression in this subgroup. This would be consistent with the previous studies demonstrating high expression levels of c-Met in about 10% of HGSOC [48]. Another c-Met inhibitor cabozantinib has demonstrated some clinical benefits in phase 2 clinical trials as a single agent in patients with platinum-resistant or -refractory ovarian cancer [49]. Interestingly, c-Met inhibition was shown to impair HR in vitro [50, 51], and it was recently shown that c-Met-mediated PARP phosphorylation confers PARPi resistance in preclinical breast cancer models [52]. However, it remains to be seen whether targeting c-Met in combination with PARP inhibition can show substantial clinical benefit in ovarian cancer.

The inhibition of cyclin-dependent kinases (CDK) has been suggested to sensitise BRCA-proficient cells to PARPis. They play a crucial role in cell cycle progression and DNA damage control, also affecting BRCA1/2 directly. CDK1 promotes mitotic progression by binding cyclinB1 [53]. Phosphorylation of BRCA1 by CDK1 is also important for activation of downstream signalling and foci formation [54]. In turn, loss of inhibition of CDK1 impairs BRCA function, creating a state of “BRCAness” [55]. It was shown in breast cancer cells that compromising CDK1 activity (by depletion or inhibition) sensitises cells to PARPis in BRCA-proficient tumours. This offers an attractive drug target, because a plethora of commercially available CDK inhibitors exists. Given CDK4/6 inhibitors, such as palbociclib or ribociclib, are already licensed for hormone-receptor positive breast cancer [52], this might facilitate early clinical translation, given the vast existing clinical experience with a comparatively similar class of drugs [56]. CDK1 inhibitors are thought to be particularly useful in combination therapy with PARPis, as this would create a state of ‘BRCAness’ [57].

The combination of PI3K inhibitors with PARPi is a well-studied and comparatively advanced approach of extending the use of PARPi. Similar to CDK1 inhibitors, PI3K inhibitors create a state of ‘BRCAness’ in BRCA-proficient cells. It has been shown that the PI3K inhibitor (BKM120) downregulates BRCA expression in cell lines and in patient-derived xenografts [58]. This was mediated by ERK signalling via the transcription factor ETS1 [58]. Other mechanisms of PI3K inhibition might be the impaired recruitment of RAD51 to sites of DNA DSBs, thus reducing HR [59]. Recent phase 1 clinical trials of combining PI3K and PARPis are promising and warrant further clinical evaluation [60, 61]. In a dose escalation trial of BKM120 in combination with olaparib, it was shown that the median duration of stable disease in patients (n = 45) without progressive disease as best overall response was 6.9 months (90% CI 5.5–7.5 months) [60].

There are promising strategies that may enhance the clinical use of PARPi, given the variety of (pre-)clinically tested combination therapies. There is particular need to increase clinical efficacy in patients with BRCAwt tumours because of the comparatively small(er) effect of PARPi in this patient cohort [10–12, 14, 17, 18, 32]. On the other hand, it will be crucial to more accurately predict PARPi efficacy in patients with HRD and (to a lesser extend) BRCA mutation carriers.

## Future prospects

Although different combination therapies could potentially enhance PARPi efficacy and circumvent resistance mechanisms, it will be pivotal to identify those patients that are most likely to benefit. Therefore, adequate predictive biomarkers will need to be identified to allow for accurate patient selection.

To screen for HRD, rather  than relying soley on the clinical response to platinum-based chemotherapy, would offer a more accurate patient stratification. This clearly depends on whether further characterisation could lead to improved treatment options for patients, as well as the availability and reproducibility of such a test. Nonetheless, it would give more patients the chance to be treated with a PARPi, and potentially additional targeted therapies.

Ongoing clinical trials are assessing the maintenance treatment of PARPi in combination with bevacizumab or checkpoint inhibitors. There are several current clinical trials investigating the use of PARPi in combination with checkpoint inhibitors in ovarian cancer (NCT03806049, NCT03574779, NCT03598270, NCT02657889, NCT02953457, NCT03522246, and NCT03737643). It remains to be seen whether those combinations show substantial clinical efficacy and improve overall survival.
